# *NTRK* insights: best practices for pathologists

**DOI:** 10.1038/s41379-021-00913-8

**Published:** 2021-09-16

**Authors:** Jaclyn F. Hechtman

**Affiliations:** Molecular Pathologist, Neogenomics 9490 NeoGenomics Way, Fort Myers, FL 33912 USA

**Keywords:** Predictive markers, Targeted therapies

## Abstract

Since the discovery of an oncogenic tropomyosin-receptor kinase (TRK) fusion protein in the early 1980s, our understanding of neurotrophic tropomyosin-receptor kinase (*NTRK*) fusions, their unique patterns of frequency in different tumor types, and methods to detect them have grown in scope and depth. Identification of these molecular alterations in the management of patients with cancer has become increasingly important with the emergence of histology-agnostic, US Food and Drug Administration-approved, effective TRK protein inhibitors. Herein, we review the biology of TRK in normal and malignant tissues, as well as the prevalence and enrichment patterns of these fusions across tumor types. Testing methods currently used to identify *NTRK1–3* fusions will be reviewed in detail, with attention to newer assays including RNA-based next-generation sequencing. Recently proposed algorithms for *NTRK* fusion testing will be compared, and practical insights provided on how testing can best be implemented and communicated within the multidisciplinary healthcare team.

## Introduction

Neurotrophic tropomyosin-receptor kinase (*NTRK*) genes encode a family of transmembrane-receptor tyrosine kinases that play an important role in neural development. The first tropomyosin-receptor kinase (TRK) fusion protein was found in 1982 in a colorectal adenocarcinoma cell line^1^. *NTRK1–3* fusions have now been identified in a number of different tumor types, including sarcomas, carcinomas, and hematologic malignancies in adults and children. The discovery of *NTRK* fusions led to the recent development of therapeutic agents that inhibit TRK fusion proteins. These agents have demonstrated good efficacy and tolerability across a wide range of *NTRK* fusion-positive malignancies and two TRK inhibitors are approved by the US Food and Drug Administration for use in patients with unresectable or metastatic *NTRK* fusion-positive cancers, agnostic of tumor type. Although *NTRK* fusions are relatively rare genomic alterations, the efficacy of TRK inhibitors creates a need to identify patients who will most likely derive benefit from these therapies^2^. Several testing methodologies to detect *NTRK* fusions are available, each with unique advantages and disadvantages. Although testing algorithms have been proposed, determining the optimal testing strategy based on available resources remains a practical challenge. Pathologists play an integral role in the identification of *NTRK* fusions and other oncogenic drivers and must be able to effectively communicate testing results and their clinical implications to the clinical oncology team managing the patients’ care.

## Trk biology: physiologic and oncogenic signaling

### Normal TRK receptor structure and function

The TRK-receptor family includes TRKA, TRKB, and TRKC (encoded by the genes *NTRK1, NTRK2*, and *NTRK3*, respectively), all of which share a highly homologous sequence and similar structural organization. The outer portion of the extracellular domain is composed of three leukine-rich regions, flanked on either side by a cysteine-rich domain. Two immunoglobulin-like regions make up the remainder of the extracellular domain, linking to the transmembrane domain and intracellular kinase domain^2,3^. In vitro studies have shown that the immunoglobulin domain closest to the transmembrane domain is sufficient for ligand binding and is important for determining binding specificity, although other regions of the extracellular domain have since been shown to also play a role in ligand binding^2,4^. The intracellular region contains five key tyrosine residues: three within the kinase-domain-activation loop and two on either side of the kinase domain that serve as docking sites for intracellular adapters and enzymes^2,5^.

TRK receptors bind neurotrophin family ligands, a group of highly homologous dimeric growth factors involved in the development and maintenance of the nervous system^6^. There are four neurotrophins present in human tissues: nerve-growth factor (NGF), brain-derived neurotrophic factor (BDNF), neurotrophin 3 (NT-3), and neurotrophin 4 (NT-4)^7^. Neurotrophin genes are initially translated as protein precursors, which are then cleaved by intracellular or extracellular proteases to generate mature neurotrophins^7,8^. Each TRK receptor preferentially binds a specific neurotrophin ligand or a pair of ligands: TRKA preferentially binds NGF, TRKB binds BDNF and NT-4, and TRKC binds NT-3. NT-3 can also bind the TRKA and TRKB receptors, although with lower affinity^6,7^. A number of TRK-receptor splice variants have been identified, which can alter the binding affinity for specific neurotrophin ligands and may interfere with downstream signaling^9^.

TRKA, TRKB, and TRKC are expressed in the peripheral and central nervous systems in adult tissues, as well as during embryonic development^10^. The specific pattern of expression of neurotrophins and TRK receptors in different areas of the nervous system plays an important role in maintaining normal neuronal balance. Similar to other receptor tyrosine kinases, ligand binding leads to kinase-domain activation, TRK-receptor dimerization, and autophosphorylation of intercellular tyrosine residues. The subsequent activation of downstream signaling pathways, including MAPK, PI3K, and PKC, promotes neuron growth, differentiation, and survival^6^.

### NTRK molecular aberrations

**Fig. 1:**
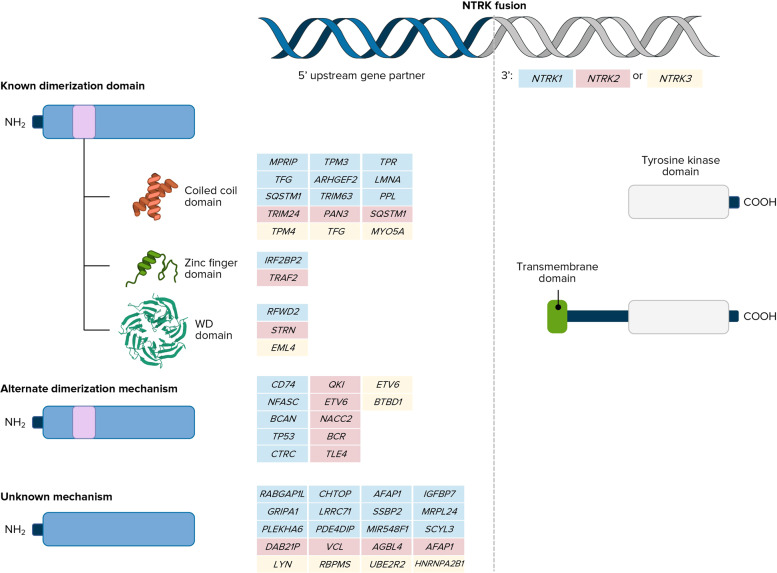
*NTRK* fusions^2^. Reprinted by permission from Springer Nature. See Ref. ^2^. Copyright 2018.

The most common oncogenic *NTRK* molecular aberrations are gene fusions that result in constitutive activation of TRK signaling. Intrachromosomal or interchromosomal gene rearrangements result in the 3′ region of the *NTRK* gene joined with the 5′ end of a fusion partner gene (Fig. 1)^2^. Over 80 different fusion gene partners have been identified to date in a wide range of tumor types^2,9^. The resulting protein contains the C-terminus of the TRK protein, including the tyrosine-kinase domain, joined to the *N*-terminus of the fusion partner. The fusion partner portion usually contains an oligomerization domain that contributes to the constitutive activation of TRK-related signaling (e.g., coiled-coil domains, zinc-finger domains, and WD domains) without ligand signaling, although alternative mechanisms of dimerization have also been reported. This constitutive activation of TRK protein ultimately leads to tumor proliferation, survival, invasion, and angiogenesis through the MAPK and PI3K pathways. The specific histology of the tumor tissue and subcellular localization of TRK receptors driven by the fusion partner can also influence downstream signaling^2^.

Beyond *NTRK* fusions, gene mutations, splice variants, and amplifications have also been explored as potential oncogenic events in a variety of malignancies. It is important to note that only fusions have been identified as actionable alterations responsive to TRK inhibitors in *NTRK1–3* genes. Somatic mutations in *NTRK* have been identified in a number of tumor types, including colorectal cancers (CRC), lung cancers, melanoma, and acute myeloid leukemias^2^. Interestingly, in vitro analysis of tumor cells harboring known *NTRK* point mutations has not shown gain-of-function to date, but instead has demonstrated impaired receptor activation and downstream signaling or no functional difference from wild-type TRK receptors^11,12^. Further studies will be needed to fully elucidate the potential role for *NTRK* nonfusion mutations in oncogenesis^13^.

TRK-receptor splice variants have also been found, including the TRKAIII splice variant in neuroblastoma. Exon skipping leads to deletion of part of the extracellular immunoglobulin-like domain normally involved in ligand binding, resulting in ligand-independent receptor activation and promotion of tumorigenic downstream signaling^14^. TRK overexpression has been reported in cancers of the breast and lung, as well as in neuroblastoma and basal-cell carcinomas. In breast cancer models, overexpression of TRKA promoted tumor-cell proliferation, migration, and invasion. In patients with neuroblastoma, TRKB overexpression was associated with higher-grade tumors and preclinical studies demonstrated responsiveness to TRK inhibitors in neuroblastoma cell lines^2^. Recently, exceptionally high expression of native, full-length TRKC (NTRK3) in the desmoplastic small round-cell tumor harboring *EWSR1-WT1* fusion has been reported to be associated with sensitivity to TRK inhibitors^15^.

### Frequency of *NTRK* fusions in oncology

*NTRK* gene fusions represent a rare genomic alteration with a widely variable distribution among different tumor types. DNA-based next-generation sequencing (NGS) screening showed an overall prevalence of 0.26% in a retrospective analysis of almost 34,000 patients and 0.28% in a similar screening program involving over 26,000 patients with cancer^16,17^. The frequency of *NTRK* fusions follows a unique pattern, with two main tumor categories (Table 1)^16,17^. First, for a select group of very rare malignancies, including secretory carcinomas of the breast and salivary gland, infantile fibrosarcomas, pleomorphic adenomas, and pediatric thyroid carcinomas, *NTRK* fusions are common (>20%) or even pathognomonic. In contrast, in the more prevalent tumor types, such as CRC, lung cancer, and invasive breast carcinomas, *NTRK* fusions are present with a much lower frequency (<1%)^18^.

**Table 1:** Frequency of NTRK fusions in patients with cancer^16–18^.

Histology	Frequency, %
Overall	0.28
Secretory breast carcinoma	92.87
Infantile fibrosarcoma	90.56
Secretory salivary gland cancer	79.68
Pleomorphic adenoma	50.47
Papillary thyroid carcinoma, pediatric	25.93
Differentiated thyroid cancer, pediatric	22.22
Inflammatory myofibroblastic tumor	17.7
Salivary gland carcinoma	5.08–5.29
Thyroid cancer	2.22–2.28
Sarcoma	0.68–1.17
Glioblastoma multiforme	0.62
Glioma/neuroepithelial tumor	0.55
Appendiceal adenocarcinoma	0.48–0.57
Melanoma	0.36–0.54
Biliary tract cancer	0.36
Cervical carcinoma	0.36
Colorectal cancer	0.26–0.35
Unknown primary	0.31
Neuroendocrine tumors	0.26–0.31
Pancreatic cancer	0.30–0.34
Cholangiocarcinoma	0.25
Lung adenocarcinoma	0.16–0.23
Invasive breast carcinoma^a^	0.08–0.13

NOS = not otherwise specified

^a^Excludes secretory breast cancer.

Over 80% of infantile fibrosarcomas and secretory carcinomas of the breast and salivary glands have *NTRK3* fusions, usually *ETV6-NTRK3*, which is pathognomonic in these rare pediatric and adult cancers. Pediatric thyroid cancers and certain gliomas have been shown to have an intermediate frequency of *NTRK* fusions, with differentiated and papillary thyroid tumors demonstrating a frequency of 22–26%, respectively^18^.

Although *NTRK* fusions are rare (<1%) in the more common types of cancer, such as CRCs and lung cancers, these aberrations demonstrate an interesting pattern related to co-occurrence with other molecular alterations. It is now known that kinase fusions including *NTRK1–3* fusions are enriched in *MLH1*-deficient colorectal carcinoma with promoter hypermethylation and wild-type *BRAF*^19,20^*. NTRK* fusions have also been identified in breast cancer specimens after progression on endocrine therapy^20^. An analysis of 76 *NTRK* fusion-positive cancers showed that co-occurrence with other oncogenic drivers is rare (*P* < 0.001) and *NTRK* fusion-positive tumors often had a lower tumor mutation burden (TMB) (*P* < 0.001)^17^. A similar study of 87 patients with *NTRK1–3* fusions also showed that these alterations were mutually exclusive with strong MAPK driver mutations in *KRAS, BRAF*, *NRAS*, and *EGFR*^16^.

This is supported by data specifically in lung cancer showing that the incidence of *NTRK* fusions is approximately 0.1–0.3% overall, but is enriched approximately 10-fold in tumors with no other identified oncogenic driver (e.g., *EGFR, ALK, ROS1*, or *RET* alterations)^16,17,21,22^. Molecular analysis of *NTRK* fusion-positive non-small-cell lung cancers showed no concurrent alterations in *KRAS*, *EGFR, ALK*, and *ROS1* or other known oncogenic drivers^22,23^.

### Targeting NTRK: where are we now and where are we going?

The discovery of *NTRK* fusions in a variety of tumor types led to development of TRK tyrosine kinase inhibitors. Two of these agents, larotrectinib and entrectinib, now have tumor agnostic approvals for patients who fulfill the following criteria^24,25^:

- An *NTRK* fusion and no acquired-resistance mutation,
- Metastatic or unresectable disease, and
- Progression on prior therapy or no satisfactory alternative treatment options.

These agents were both investigated in basket studies that enrolled different types of *NTRK* fusion-positive tumors. In a pooled analysis, larotrectinib demonstrated an objective-response rate (ORR) of 78% and median progression-free survival of 36.8 months. Responses were durable, with median duration of response reached and over 66% of responses maintained after two years. Larotrectinib was also active in patients with brain metastases, with an ORR of 71%^26^. A similar analysis of entrectinib in *NTRK* fusion-positive cancers showed an ORR of 63.5%, median duration of response of 12.9 months, median progression-free survival of 11.2 months, and intracranial ORR of 50.0%^27^.

As with other tyrosine-kinase inhibitors, resistance can develop over time in patients receiving TRK inhibitors. Point mutations in the *NTRK*-kinase domain have emerged as an important mechanism of resistance, including G667C in *NTRK1* and G696A in *NTRK3*^28^. Activation of the MAPK signaling pathway through hot-spot mutations in *KRAS* and *BRAF* and amplification of *MET* have caused acquired resistance to first-generation TRK inhibitors in gastrointestinal cancers^29^. This has led to investigations of second-generation TRK inhibitors as a strategy to overcome resistance, with selitrectinib and repotrectinib demonstrating promising early activity in this setting^30,31^.

### The nuts and bolts of *NTRK* fusion testing

In order to accurately identify patients who may benefit from TRK-targeted therapies, *NTRK* fusion testing needs to be done consistently and with sensitive and specific methodologies. There are several assays currently available to identify *NTRK* fusions in tumor samples, including immunohistochemistry (IHC), fluorescent in situ hybridization (FISH), reverse transcriptase–polymerase chain reaction (RT-PCR), and NGS-based analysis (Table 2). Each of these testing strategies has advantages and disadvantages that must be taken into consideration for a given tumor specimen. In addition to variability in sensitivity and specificity, practical differences regarding access, cost, and turnaround time can play an important role in molecular testing decisions^32,33^.

**Table 2:** Advantages and limitations of *NTRK* fusion assays^16,32,33^.

	Material required	Approximate turnaround time	Sensitivity	Specificity	Other considerations
**IHC**	1 unstained slide	1 day	• 96.2% for *NTRK1* • 100% for *NTRK2* • 79.4% for *NTRK3*	• 81.1% • Variable based on tumor type	• Relatively inexpensive • Interpretation must take tumor histology into account
**FISH**	3 unstained slides (1 for each *NTRK* gene)	1–3 days	• Highly sensitive • Depends on breakpoints	High specificity, yet cannot clarify structural variants of uncertain significance	• Relatively inexpensive • Useful when high suspicion of *ETV6-NTRK3* fusions
**RT-PCR**	1 µg of RNA (~50 000 cells)	1 week	• Variable (see notes) • Need decent RNA quality	• High	• Relatively inexpensive • Both involved genes and exons must be included in primers
**DNA-based NGS**	Approximately 250 ng of DNA, but depends on assay (~50 000 cells)	2–4 weeks	• 96.8% for *NTRK1* • 76.9% for *NTRK3*	• 99.86% • Dependent on whether structural variant results in transcribed fusion	• Relatively expensive, difficult to tile *NTRK3* kinase domain introns, need decent tumor purity • Can simultaneously assess point mutations, other fusions, tumor mutation burden, copy number changes, microsatellite instability status
**RNA-based NGS**	Approximately 200 ng of RNA, but depends on assay (~10 000 cells)	2–4 weeks	• 95.3%; dependent on RNA quality	100%	• Relatively expensive • Can assess other fusions and oncogenic transcripts across multiple genes, as well as splice variants
**DNA/RNA NGS**	10 ng to 40 ng of RNA (>20% tumor content)	2–4 weeks	98% to 100%	96–100%	• Relatively expensive • Can assess other aberrations listed for DNA and RNA NGS assays above

Abbreviations: FISH fluorescent in situ hybridization, IHC immunohistochemistry, NGS next generation sequencing, RT-PCR reverse transcription polymerase chain reaction.

### Immunohistochemistry

Use of IHC to assess TRK-fusion protein expression is widely available in clinical laboratories, relatively inexpensive, and has a rapid turnaround time, typically within 24 h. Commercially available IHC clone EPR17341 is the most frequently used IHC antibody. It is monoclonal, and the epitope recognizes a sequence homologous between the 3 *NTRK* genes in the C-terminus of the protein, which, unlike the 5′ end, is retained in fusion proteins^32,34^. While purchasing this antibody by itself requires a validation for clinical use within a lab, as it is a laboratory-developed test, an in vitro diagnostic product with this clone is also available^35^. The in vitro diagnostic product requires a verification rather than a validation, which is more feasible for labs without a significant number of known *NTRK* fusion-positive tumors available for validation. Immunohistochemistry requires one unstained tumor slide and is less dependent on tumor purity compared with other biomarker testing methodologies. TRK staining in ≥1% of tumor cells is considered *NTRK* fusion-positive to increase sensitivity, as *NTRK3* fusion-positive tumors may show focal or weak expression. Pan-TRK IHC has demonstrated a sensitivity of 96.2 and 100% for *NTRK1* and *NTRK2* fusions, while a lower sensitivity of 79.4% was observed for *NTRK3*^16^. In addition, when staining is present in tumors with *NTRK3* fusions, it may be focal and weak. This lower sensitivity for *NTRK3* fusions suggests that alternative testing methods should be considered to evaluate tumors characterized by *NTRK3* fusions when histology is suggestive, including secretory carcinomas and infantile fibrosarcoma^32^.

**Fig. 2:**
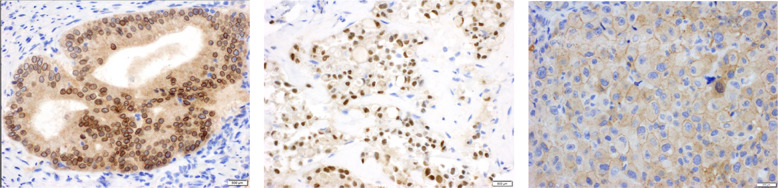
Variable IHC staining patterns for *NTRK* fusions. Patterns of pan-TRK IHC expression in *NTRK* fusion-positive cancers. **A** A colorectal carcinoma with *an LMNA-NTRK1* fusion demonstrates diffuse cytoplasmic expression with accentuation of the nuclear member with pan-TRK immunohistochemistry. The *LMNA* gene encodes nuclear lamin, which localizes to this area of accentuation. **B** Pan-TRK immunohistochemistry performed on secretory carcinoma reveals diffuse cytoplasmic and strong (3+) nuclear expression. *ETV6* encodes a transcription factor that localizes to the nucleus. Although this case demonstrates diffuse staining, *ETV6-NTRK3* fusion-positive cancers often show weak and/or focal pan-TRK expression by immunohistochemistry. **C** A melanoma with a *TRAF2-NTRK2* fusion demonstrates diffuse cytoplasmic and membranous expression on pan-TRK immunohistochemistry. *TRAF2* encodes TNF-receptor-associated factor 2, which localizes to the cell membrane.

Although cytoplasmic staining is the typical pattern for physiologic TRK expression by IHC, the pattern of IHC staining in *NTRK* fusion-positive cancers can vary based on the localization pattern associated with the fusion partner. Cytoplasmic, nuclear, perinuclear, and membranous staining have all been observed, requiring pathologists to be familiar with the variable staining patterns in order to improve the accuracy of *NTRK* fusion testing (Fig. 2)^16^.

Another important consideration is differences in specificity based on tumor type. In a recent analysis of 87 *NTRK* fusion-positive cancers, specificity of pan-TRK IHC was 100% for tumors of the colon, lung, thyroid, pancreas, and appendix, as well as in patients with melanoma. However, specificity was lower in breast cancers (82.1%) and salivary gland tumors (52%), which may be due to cytoplasmic IHC staining. Sensitivity and specificity are also low for sarcomas (80% and 74.4%, respectively), as TRK proteins are expressed in nonneoplastic neural and smooth-muscle tissue. Other methods of *NTRK* fusion testing should be considered in these tumor types^16^. Even in tumors exhibiting a high sensitivity and specificity for IHC-based testing, confirmatory testing with nucleic acid-based analysis should be performed when feasible^2^.

### Fluorescence in situ hybridization

**Fig. 3:**
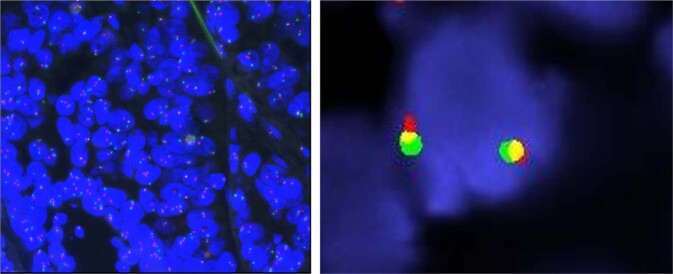
*NTRK* FISH analysis with break-apart probes. **A** FISH positive for *NTRK* gene rearrangement. **B** FISH negative for *NTRK* gene rearrangement.

Fluorescent in situ hybridization is a highly sensitive DNA-based assay that identifies oncogenic fusions using either break-apart probes or fusion probes (Fig. 3). Fluorescent in situ hybridization has historically been the standard method for detection of gene rearrangements, including *ALK*, *ROS1*, and *RET* rearrangements or fusions in NSCLC. This testing method is relatively inexpensive and widely available in clinical laboratories, with a short turnaround time of typically 1–3 days. Formalin-fixed paraffin-embedded (FFPE) tissue can be used and testing is generally reliable even in samples with low tumor purity^32^. Break-apart FISH rather than fusion FISH is typically used to assess *NTRK* fusions because there are many known 5′ partners to assess. Each FISH assay evaluates a single *NTRK* gene, so three separate slides are required to assess *NTRK1, NTRK2*, and *NTRK3* fusions. Development of FISH multiprobes that can simultaneously target all 3 *NTRK* genes will likely reduce the time and resources needed for testing^36^.

Break-apart FISH can assess whether or not a gene is rearranged. Break-apart probes do not allow identification of the fusion partner involved in *an NTRK* rearrangement, nor are they capable of determining whether a known rearrangement results in a functional fusion protein^34,36^. While FISH is highly sensitive for fusions with canonical breakpoints, there is a potential for false negatives if the fusion breakpoint involves noncanonical sites. Short inversions and intrachromosomal rearrangements, which are common in *NTRK1* fusions, result in short split length using break-apart probes and can result in false negatives^32,34^.

### Reverse transcription polymerase chain reaction

Reverse-transcription polymerase chain reaction detects gene-fusion RNA transcripts and can be either qualitative or quantitative. This technique is relatively inexpensive and the results take approximately one week. Reverse-transcription polymerase chain reaction assays require approximately 1 µg of RNA (roughly 50,000 tumor cells) and the reliability is highly dependent on the quality of the RNA sample. An important limitation of RT-PCR in *NTRK* fusion testing is the requirement to know both the fusion partner and the exon breakpoints when designing the primers^32^. Over 80 different fusion partners have been identified in oncogenic *NTRK* fusions and there is significant variability in the breakpoints and exons involved, limiting the utility of RT-PCR in clinical practice^9,32^. This approach can be considered for tumor histologies with known fusion partners, such as detection of *ETV6–NTRK3* fusions in infantile fibrosarcoma or secretory breast cancers^36^.

### Next-generation sequencing

Next-generation sequencing analysis provides highly sensitive and specific detection of *NTRK* gene fusions in tumor samples. A major advantage is the ability to simultaneously evaluate many potential oncogenic drivers, including *NTRK* fusions with novel fusion partners. The precise amount of genetic material required for testing varies based on the platform used, but all NGS testing requires high-quality DNA or RNA. Next-generation sequencing is associated with higher cost compared with IHC and FISH testing and is not as widely available. The results generally take 2–4 weeks, which can be an important consideration for management of patients with significant tumor burden^32^.

### Laboratory considerations and logistics for NGS analysis

A number of NGS platforms are currently available and each differs with regard to the amount of genetic material required, the number and types of genes evaluated, and the depth of coverage of the target genomic regions^32^. A typical NGS workflow begins with extraction of DNA or RNA from a tumor sample. This genetic material is then assessed for quality and quantity and the DNA/RNA library is prepared. Target enrichment with either amplicon-based or hybridization-capture-based methods is performed, followed by sequencing. Once sequencing is complete, a bioinformatics process begins that involves sequence alignment with a human reference genome, quality control, and variant calling to identify alterations in the tumor genetic sequence. Once mutations, amplifications, fusions, rearrangements, deletions, etc., have been identified, these alterations are annotated and a report is generated to convey the findings to the healthcare team^37^.

#### DNA-based NGS

DNA-based NGS assays can simultaneously examine point mutations, amplifications, deletions, fusions, microsatellite-instability (MSI) status, and TMB status. This can be particularly useful in determining which patients need further biomarker testing, as *NTRK* fusions are generally mutually exclusive with “driver” *MAPK* alterations such as other kinase fusions, *RAS* mutations, and *BRAF* V600E mutations^32^. DNA-based NGS is also useful to monitor for *NTRK* mutations in patients receiving TRK inhibitors, as this is an important mechanism of resistance to these agents^34^. As with FISH testing, DNA-based NGS identifies DNA-level genomic rearrangements that may or may not result in a functional fusion protein. As a result, further testing using RNA-based analysis may be needed to confirm a positive finding^32^.

DNA-based NGS is typically either hybridization-capture-based or amplicon-based. DNA hybridization-capture-based NGS has been used to assess *NTRK* fusions. It requires approximately 250 ng of high-quality DNA extracted from FFPE tumor tissue, although the precise nucleic acid requirements differ for each assay^32^. There are several different methods available for target enrichment, including amplicon-based and hybridization-capture-based approaches. Amplicon-based methods use PCR primers to amplify the genes of interest and are appropriate for detection of point mutations and small insertions and deletions, but are not ideal for assessment of gene fusions that usually involve intronic breakpoints. In addition, amplicon-based methods require knowledge of both fusion partners for accurate primer design. Hybridization capture uses probes in a nonbiased approach to allow deep sequencing of exons and identification of point mutations, insertions/deletions, and copy number variations. In addition, hybridization-capture-based target enrichment can include additional probes specific for kinase-domain introns of target oncogenic fusions to allow detection of these rearrangements with unknown fusion partners as well^34^.

In an analysis of 87 *NTRK* fusion-positive cancers, DNA-based NGS demonstrated a specificity of 99.86% and an overall sensitivity of 81.1%. This approach is highly sensitive in detection of *NTRK1* fusions (96.8%), but shows lower sensitivity for *NTRK3* fusions (76.9%) and did not detect any of the 4 *NTRK2* fusions present in this study^16^. Several of the hybridization-capture-based NGS platforms include capture probes specific for introns of *NTRK1, NTRK2*, and *ETV6*, a common fusion partner of *NTRK3*, but do not include probes targeting *NTRK3* intronic regions^34^. The intronic regions adjacent to the exons that encode the kinase domain of *NTRK3* are too large to reasonably cover, as this would compromise coverage of other target genes within the NGS panel. In addition, some of the intronic regions of *NTRK3* are highly repetitive and result in sequences that cannot be accurately mapped back to the appropriate intron. As a result, DNA-based NGS shows reduced sensitivity for *NTRK3* fusions and could miss those that do not involve *ETV6*^16,34^.

#### RNA-based NGS

RNA-level analysis of *NTRK* fusions removes the complication of intronic regions associated with DNA-based NGS, allowing confirmation of transcribed fusions. In addition, the precise fusion partners and exons involved in the fusion transcript are identified and multiple genetic alterations can be assessed simultaneously. RNA-based NGS requires approximately 200 ng of RNA, although this differs depending on the assay, and RNA quality is vital to the integrity of the results. RNA degradation in FFPE samples is a significant problem, particularly with older samples^32^. Specialized reagents and careful handling are necessary, as well as assessment of RNA quality prior to analysis^38^.

After RNA is extracted from tumor tissue, the RNA library is converted to cDNA. Amplicon-based panels then use standard multiplex PCR or anchored multiplex PCR to amplify the sequences of interest^32^. If standard multiplex PCR is used, both fusion partners must be known to design appropriate primers^32,39^. Anchored multiplex PCR adds a sequencing adapter to each end of the cDNA, so during PCR, the *NTRK*-specific primer binds to the *NTRK* region and a universal primer hybridizes to the adapter sequence downstream of the unknown fusion partner. This allows identification of *NTRK* fusions with novel fusion partners, improving sensitivity^22,32,40^. Hybridization-capture-based approaches can also be used in RNA-based NGS, requiring only one fusion partner to be known^41^.

#### Hybrid DNA/RNA panels

New NGS platforms are now providing simultaneous analysis of DNA-level and RNA-level genomic aberrations from the same FFPE tumor sample. The DNA and RNA libraries are prepared separately, then the cDNA created from the extract RNA, as well as the DNA, is pooled for a combined sequencing run. Many different oncogenic drivers can be investigated at the same time with very little genetic material, a distinct advantage when tumor tissue is limited^32^. Current assays use either hybridization-capture-based or amplicon-based target enrichment, with amplicon-based enrichment requiring knowledge of both fusion partners, as mentioned previously^42,43^.

### Circulating tumor DNA analysis

Assessment of circulating-tumor DNA (ctDNA) provides a noninvasive approach to monitor tumor biology in patients with cancer. For patients with *NTRK* gene fusions receiving TRK-inhibitor therapy, ctDNA-based analysis can allow monitoring for tumor recurrence or progression on treatment. Next-generation sequencing of these samples can identify resistance mutations and select patients who may be eligible for clinical trials investigating emerging next-generation TRK inhibitors. Sensitivity can be an issue with ctDNA analysis, as detection of genetic alterations requires adequate tumor cell shedding for detection in the circulation^32^. In addition, a study using DNA-based NGS analysis of ctDNA samples from patients with lung cancer showed a sensitivity of only 54.2% for detection of *ALK* fusions, suggesting that oncogenic fusion detection may be challenging in ctDNA samples^44^.

### Who should be tested? A look at the algorithms

The efficacy of TRK inhibitors across tumor types makes a clear argument for *NTRK* fusion testing in patients with advanced cancers^2^. There is currently no role for TRK inhibitors in early-stage disease, so screening is not a priority in patients with localized disease, unless *NTRK* fusions are pathognomonic. Multiple testing algorithms for *NTRK* fusions have been proposed, making determination of the optimal strategy challenging. Testing decisions should ultimately be influenced by the tumor type and resources available, including the quality and quantity of biopsy material and availability of testing methods. Although IHC and FISH are both associated with unique challenges related to fusion testing, these testing methods should be strongly considered when access to NGS is limited^34^.

### Histology-based triage

Investigators at Memorial Sloan Kettering Cancer Center recently published a suggested algorithm for *NTRK* fusion testing that begins with histology-based triage. In histology-based triage, tumors are separated into groups of high and low probability of *an NTRK* fusion based on the tumor histology. Those with a high probability, such as tumors with histologic features of infantile fibrosarcoma or secretory carcinoma, should undergo automatic *NTRK* fusion testing. Within this group, patients with sarcoma should receive FISH or RNA-based fusion testing (based on reduced specificity and sensitivity for IHC analysis)^34^. Studies are now identifying unique sarcoma subtypes that may also be more likely to harbor *NTRK* fusions, including uterine spindle-cell sarcomas with features similar to fibrosarcomas^45^. Patients with carcinoma should have pan-TRK IHC, with negative results followed up with FISH or RNA-based testing. Patients with sarcomas that have a lower probability of having *an NTRK* fusion should be evaluated using RNA-based NGS testing, while those with other tumor types can undergo routine screening or move on to genomic-based triage^34^.

### Pan-cancer screening

For tumors that have a low likelihood of *an NTRK* fusion (e.g., lung cancer, breast cancer, CRC, pancreatic cancer, and melanoma), the European Society of Medical Oncology Translational Research and Precision Medicine Working Group recently recommended using NGS-based testing, when available, with RNA testing and IHC confirmation for positive cases for all patients with advanced cancers. If NGS is not available, IHC-based mass screening is recommended^46^. While these approaches increase the probability of identifying patients who may benefit from TRK inhibitors, careful consideration of cost and availability of testing is needed. Pan-cancer screening using a single-analyte assay is an inefficient approach for detection of rare biomarkers like *NTRK* fusions^34^.

### Genomic-based triage

When pan-cancer screening is not feasible or economical, genomic-based triaging can help identify cases with the highest priority for further *NTRK* fusion testing. As mentioned previously, *NTRK* fusions are often mutually exclusive with oncogenic driver alterations, including *KRAS, NRAS, BRAF, EGFR, ALK, RET, ROS1*, *KIT*, and *PDGFRA*, and are associated with MSI-high status (specifically, with *MLH1* promoter hypermethylation). Thus, tumors lacking these common oncogenic drivers represent good candidates for *NTRK* fusion testing. The Memorial Sloan Kettering Cancer Center algorithm suggests that patients with lung carcinoma who have no oncogenic drivers identified during panel-based NGS testing and a low TMB should be considered for IHC or RNA-based NGS testing. Those with CRC that are negative for traditional oncogenic alterations and have an MSI-high status should also be screened for *NTRK* fusions^34^. *NTRK* fusion testing should also be considered in patients with hormone-receptor-positive advanced breast cancer after progression on endocrine therapy. A recent study showed that these tumors may be enriched for kinase fusions as a mechanism of resistance, including *NTRK* fusions^47^.

### Role of pathologists in clinical decision-making

Pathologists play a critical role in the diagnosis and assessment of patients with cancer. Tissue stewardship and prioritization of testing when tumor tissue is limited are important elements in patient care, ensuring that the appropriate biomarkers are evaluated to inform treatment selection^48^. In addition, accurate reporting and communication of biomarker findings is critical to ensure that appropriate treatment decisions are made and patients receive optimal care. Current recommendations from the Association for Molecular Pathology, American Society of Clinical Oncology, and College of American Pathologists indicate that NGS reporting should include tier-I through tier-III findings (variants of strong clinical significance, variants of potential clinical significance, and variants of unknown significance). It is currently not recommended to include tier-IV findings (benign or likely benign variants). Reports should include positive findings and pertinent negative findings with a strong clinical significance^49^.

For *NTRK* alterations, clear diagnostic reports are needed that provide annotations on the specific NTRK abnormality found and whether that alteration is actionable. Patients with *NTRK* amplifications and mutations are not currently eligible for TRK-inhibitor therapy; only those with *NTRK* gene fusions are eligible^24,25^. If *an NTRK* gene rearrangement is detected on DNA-based NGS analysis, the report should specify whether the rearrangement is a fusion, whether it is predicted to be in-frame, and whether the kinase domain is involved. Ideally, reflex testing should be performed when initial testing demonstrates a rearrangement that is not predicted to form a canonic fusion. For example, FISH or RNA-based testing should be performed if IHC testing is negative in tumor histologies with a high likelihood of *NTRK* fusions^34^. Likewise, confirmatory testing with IHC should be performed to detect functional fusion protein expression in patients with positive NGS-based fusion testing^46^. If reflex testing is not part of the standard protocol, the pathology report should specify that additional testing is recommended and which type of assay would be most useful.

Identifying and targeting *NTRK* fusions continues to change the treatment landscape for patients with a variety of rare and common malignancies. Appropriate, accurate testing for these rare genetic alterations is essential to inform treatment selection and ensure that eligible patients are receiving the most effective therapies available to them. Pathologists are at the center of this evolving paradigm, identifying *NTRK* fusions and other actionable biomarkers and communicating these findings to other members of the multidisciplinary healthcare team. By carefully implementing fusion-testing algorithms and selecting optimal testing methodologies, pathologists can greatly improve the utility of TRK inhibitors in patients with cancer and as a result, improve patient outcomes.
